# Testing the METUX Model in Higher Education: Interface and Task Need–Satisfaction Predict Engagement, Learning, and Well-Being

**DOI:** 10.3389/fpsyg.2021.631564

**Published:** 2021-02-24

**Authors:** Lucas M. Jeno, Åge Diseth, John-Arvid Grytnes

**Affiliations:** ^1^Department of Education, University of Bergen, Bergen, Norway; ^2^Department of Biological Sciences, University of Bergen, Bergen, Norway

**Keywords:** higher education, self-determination theory, METUX, achievement, technology

## Abstract

The main aim of this study is to test the validity of the Motivation, Engagement, and Thriving in User Experience (METUX) model (Peters et al., 2018) in higher education. We propose a process model in which we investigate how the need-satisfaction of digital learning tools within the interface sphere and task sphere accounts for engagement, learning, and well-being. A total of 426 higher education students drawn from two subsamples participated in this cross-sectional study. A structural equation model shows that interface autonomy and competence satisfaction positively predict task autonomy and competence. Task competence, in turn, negatively predicts focused attention and positively predicts perceived usability and well-being. Task autonomy positively predicts perceived usability and reward. Based on our results, we provide some initial support for the METUX model in higher education. However, more validation work is needed to improve the scale that measures need-satisfaction in the interface and task spheres. Moreover, we find no support for the effect of task sphere on learning. Further investigations are needed into how METUX can be used in domain- and situation-specific contexts to account for increases in engagement, learning, and well-being. Finally, future studies need to include all aspects of the METUX model in order to fully test its validity.

## Introduction

Technological devices (i.e., smartphones, tablets, personal computers) have become ubiquitous for higher education students in everyday life and for learning purposes. Recent reports suggest that 97% of the student population in Norway owns some form of technological device (Slettemeås and Kjørstad, 2016). Similar results are found in the United States and the United Kingdom (Brooks and Pomerantz, 2017). Technological devices have the benefits of providing students in higher education opportunities to take notes, access multimedia content, produce content, and retrieve information (Danish and Hmelo-Silver, 2020). They also enable students to collaborate with peers, both asynchronously and synchronously. Moreover, technological devices also give students access to thousands of digital learning tools, such as applications, software, and online resources that are developed for learning purposes. A report estimated that there are 500,000 educational apps available through App Store and Android for learning (Lowrie and Jorgensen, 2015), and this number is increasing. Thus, technological devices provide several opportunities to support learning through not only formal educational contexts but also in informal contexts through peer collaboration on assignments, retrieval of information, or communication. For instance, previous research has shown that academic achievement is positively predicted by the use of social networking sites for academic purposes such as Facebook (Wang et al., 2012) and Twitter (Rinaldo et al., 2011). This effect is also apparent in a meta-analysis (Marker et al., 2018).

The way in which the interface and multimedia of such digital learning tools are designed impacts student learning, engagement, and well-being (Peters, 2014). Based on this, Peters et al. (2018) developed a model grounded on the motivational theory of Self-Determination Theory (SDT). This model describes the user experience of digital learning tools, and it is called “motivation, engagement, and thriving in the user experience” (METUX; Peters et al., 2018). The METUX model provides an interesting framework for analyzing the extent to which the content (i.e., design) and functionalities of the digital learning tools enhance or hinder engagement, learning, and well-being.

These issues are particularly crucial for students within higher education because they are more independent in their learning. They have more choices regarding when, how, and with whom they learn, and the use of technological devices is often an important part of their learning experiences. Hence, it is useful to investigate how their usage of digital tools is related to their engagement, learning, and well-being as accounted for by the METUX model. Thus, the main aim of our study is to investigate how students in a higher education context experience using digital learning devices as described in the METUX model. A further aim is to investigate how the METUX model predicts engagement, learning, and well-being. In short, the present study will provide an initial test and validation of the METUX model in higher education.

### The METUX Model Within SDT

The theoretical propositions of the METUX model are grounded on the theoretical tenets of SDT. From an SDT perspective, humans have three universal and basic psychological needs for autonomy, competence, and relatedness (Ryan and Deci, 2017). The satisfaction of these basic needs is assumed to be necessary for optimal human functioning, whereas the thwarting of the needs is assumed to result in maladjustment and non-optimal functioning (Vansteenkiste and Ryan, 2013). Autonomy refers to the need to feel self-endorsement and volition in relation to one's actions and experiences. The need for autonomy is satisfied when students feel that they have meaningful choices for their behaviors, when pursuing their authentic interests, or when experiencing congruency and harmony in relation to a behavior. Competence refers to the need to feel effectance and mastery in interacting with one's environment. The need for competence is satisfied when students experience optimal challenges for their level, receive feedback, proper scaffolding, or structure, or master tasks in different life contexts. Finally, relatedness refers to the need to belong, feel cared for by significant others, and contribute and give to others. The need for relatedness is satisfied when students experience social connection, a feeling of importance to others or to a social organization, or a sense of belongingness through contributing to a group.

The manifestation of basic need-satisfaction of autonomy, competence, and relatedness is higher engagement, growth and learning, and psychological wellness in relation to an activity or situation. In a learning context, satisfaction of basic psychological need-satisfaction means that students experience more engagement for a learning activity. The result is more conceptual learning and, at the same time, experience of more psychological wellness due to optimal functioning (Deci et al., 1991; Ryan and Deci, 2017).

Based on SDT, Peters et al. (2018) developed the METUX model for technology. As conceptualized within METUX, interaction with technology could impact engagement, learning, and psychological well-being through different experiences and spheres, that is, the psychological experience of interacting with features and interface design and using different digital learning tools can satisfy students' psychological needs for autonomy, competence, and relatedness when using these different technologies (Ryan and Deci, 2017). The METUX model assumes that students' perceived need-satisfaction is a mediator between their experience of using digital learning tools and their engagement, learning, and well-being in learning situations. We explain the METUX model in detail below.

### The Need-Satisfying Spheres of Technology Within METUX

The METUX model proposes the necessity of experiencing need-satisfaction in six different spheres in order for technology to positively impact engagement, learning, and well-being. The METUX model assumes that interaction with digital learning tools produce specific outcomes corresponding to each specific sphere (Peters et al., 2018). Hence, the satisfaction of the basic psychological needs in each sphere mediates the effect on the outcomes corresponding to the specific spheres. The six different spheres of experience within learning technology in the METUX model are adoption, interface, task, behavior, life, and society and are described below.

1. The *adoption sphere* consists of becoming aware of and acquiring new technology. For example, a student's awareness of an instructional video on the online course platform Coursera or a learning management system (LMS) app may result in the adoption of this digital learning tool in order to support the learning task. According to the METUX model, the extent to which the technology is adopted and used over time depends on the motivation of the student for acquiring it. This motivation may be either autonomous (behaviors enacted out of volition and choice) or controlled (behaviors enacted out of pressure or contingent on self-worth).
2. The *interface sphere* consists of interacting with the technology's interface during use, for instance, a student's experience of controls and navigation, display, and aesthetic within Coursera or LMS app when using it for a learning task. When students interact with a digital learning tool, the satisfaction of the basic psychological needs, *via* the user interface, predict usability, engagement with technology, and user satisfaction.
3. The *task sphere* consists of engaging in a technology-specific task. An example of this is the extent to which students experience that Coursera or an LMS app support their learning by means of features and functionalities in these digital tools. When students engage with tasks, the satisfaction of basic psychological needs, *via* engagement with technology-specific tasks, predicts engagement with the task and user satisfaction.
4. The fourth sphere is *behavior*, which consists of engaging in a behavior that the technology is intended to support. For example, students experience that the Coursera or LMS app increases learning, which is what it is intended to do. When students engage in a behavior that the digital learning tool is intended to increase, the extent to which students actually learn as a result of using the digital learning tool depends on the need-satisfaction when learning.
5. The user's overall *experience of life sphere* lies beyond the technology. For example, students may learn skills such as mindfulness, mental health awareness, and sleep improvement within a Coursera course that increases their well-being beyond the behavior sphere, that is, learning about mindfulness and doing mindfulness may increase learning of the practice and theory (behavior sphere), plus practicing mindfulness may increase life satisfaction and positive affect (life sphere) because the student becomes more aware and attentive of moment-to-moment situations, which may in itself be need-satisfying.
6. The *society sphere* consists of the experience of all members of society, beyond the technology. An example of this is a well-designed instructional video in Coursera on environmental conservation. After interacting and learning the content of the video, the viewer may display more environment-friendly behaviors, thus reducing pollution of the sea, land, and air.

### Need-Satisfying Spheres and Their Relation to Engagement, Learning, and Well-Being

As previously mentioned, the METUX model has yet to be validated, and there is no empirical support for its effectiveness in a higher education context. However, Peters et al. (2018) provided some preliminary findings in support of the METUX model in their appendix. For instance, need-satisfaction in the interface sphere positively predicted need-satisfaction in the task sphere and, in turn, need-satisfaction in life and technology satisfaction. Despite the lack of direct support for the different spheres within the METUX model and their relation to engagement, learning, and well-being, there have been studies in areas of digital learning tools and technology in general. Previous research has also investigated how support of need-satisfaction enhances engagement, learning, and well-being, which, indirectly, may support our line of reasoning.

#### Engagement

In particular, research has shown that interaction with digital tools in learning is related to the quality of user experience (i.e., engagement) (O'Brien and Toms, 2010; O'Brien, 2016). Mobile apps, as compared to traditional pen and paper, increase engagement both when measuring the use of the app and with open-ended questions (Hartman et al., 2019). Ford et al. (2012) show that whole-body games designed to satisfy the needs for autonomy, competence, and relatedness promoted user engagement among kindergarten children. An experimental study by Peng et al. (2012) also shows that video games in which autonomy and competence features were switched on, as opposed to off, increased the participants' game enjoyment, game recommendation and ratings, and motivation for future play. Finally, a study by Ijaz et al. (2020) found that open world platforms in virtual reality, compared to static user interface platforms, enhanced the participants' autonomy, enjoyment, and feelings of immersion in a randomized experiment.

#### Learning

Technology and digital learning tools have the potential to support student learning and achievement by means of anytime and anywhere information-seeking, social connectivity, and collaboration and as cognitive tools (Peters, 2014; Danish and Hmelo-Silver, 2020). Accordingly, Fathali and Okada (2017) found that need-satisfaction of competence predicted achievement and intention to continue using the technology among second-language learners using a web-based electronic portfolio. Furthermore, randomized experiments on biology students have shown that need-satisfying mobile apps enhance students' achievement compared to a textbook condition (Jeno et al., 2017a, 2019b). Finally, a meta-analysis has shown that technology, on average, has a positive effect on achievement among higher education students (Schmid et al., 2014).

#### Well-Being

Digital learning tools can potentially not only impact students' well-being negatively through peer comparison, decreased inhibition of anti-social behavior, and fear of missing out but also impact positively through active engagement with peers, access to mental health intervention programs, and arenas for personal disclosure (Lattie et al., 2019). In one of the first studies on technology within SDT, Ryan et al. (2006) found that experiences of need-satisfaction of autonomy and competence in a video game enhanced the participants' well-being from pre-test to post-test. Research conducted in an online setting has found that online need-satisfaction positively affects well-being indicators among children (Shen et al., 2013) and students (Wang et al., 2015) over and above need-satisfaction in daily-life. Similar results have been found in the educational context. For instance, Jeno et al. (2019a,b) found that a mobile app reduced the negative affect in a controlled experiment in higher education. This finding was presumably due to need-satisfaction of autonomy and competence inherent within the mobile app. In a three-wave study on mobile phone usage, Hong et al. (2020) found that autonomy need-dissatisfaction was related to problematic mobile phone usage.

Taken together, previous research findings give reasons to expect that need-satisfying digital learning tools enhance user engagement due to the provision of meaningful choices or ability to pursue interesting and valuable content and information. Previous research also supports the assumption that need-satisfying digital learning tools enhance learning due to the provision of structure, effectance-relevant feedback, optimal challenges, and interesting and novel information. Finally, well-being is enhanced when digital learning tools provide support for basic psychological needs either through features, tasks, or behaviors. Conversely, well-being is reduced when basic psychological needs are thwarted.

In summary, these findings provide some indirect support for the basic tenets of the METUX model in which the basic psychological needs mediate the relation between the interface and tasks within digital learning tools and engagement, learning, and well-being.

### The Present Study

A primary aim of the present study is to provide empirical support for the METUX model. As mentioned above, METUX proposes six spheres that might impact user experience. In this study, we only employ four of the six spheres, namely, the interface, task, behavior, and life spheres. We exclude the adoption sphere because we explicitly measure the use of digital learning tools for learning purposes. Hence, we assume that the students are already aware of the digital learning tools and have already acquired them due to some formal or informal learning purposes. We also exclude the societal sphere because this study is cross-sectional in nature, and thus we could not, based on our cross-sectional study design, infer that the digital learning tool can impact the society in any meaningful way. Moreover, we measured only autonomy and competence. Relatedness was excluded due to the difficulty of knowing whether many of the idiosyncratic digital learning tools had elements of relatedness in the technology design. In order to remove this potential confounding effect, we decided to exclude the satisfaction of relatedness.

**Figure 1:**
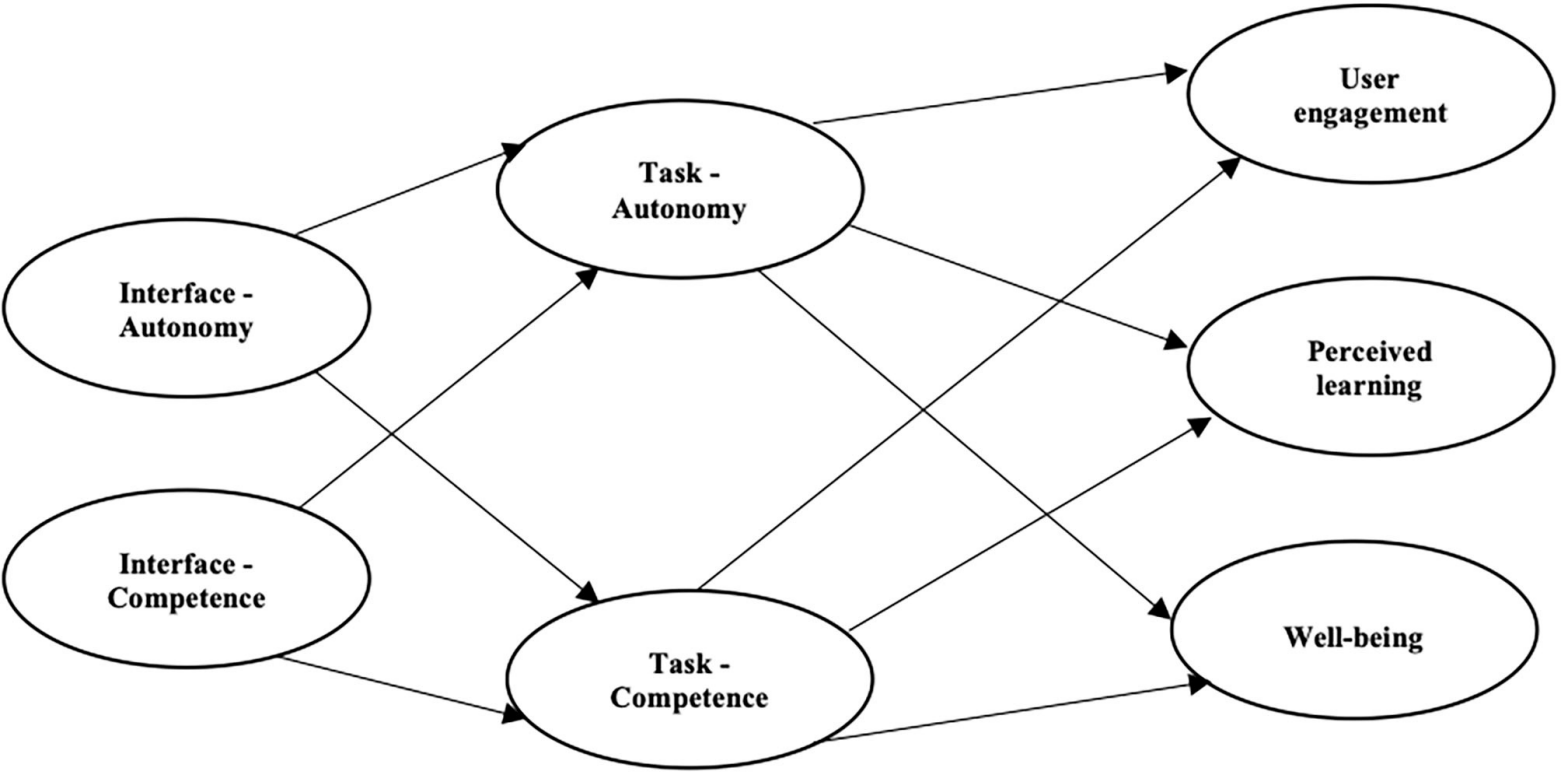
Structural Equation Model of autonomy and competence at the interface and task levels as predictors of engagement, learning, and well-being. We expect the interface sphere to positively predict the task sphere and indirectly predict increases in user engagement, perceived learning, and well-being. We expect a positive relation between the task sphere and user engagement, well-being, and perceived learning.

An additional aim is to investigate how need-satisfaction within different spheres in the METUX model account for increases in engagement, learning, and well-being. To investigate this, we test a model (Figure 1) predicting that need-satisfaction of autonomy and competence in the interface sphere will positively affect need-satisfaction of autonomy and competence in the task sphere. Task sphere need-satisfaction will, in turn, positively predict user engagement and learning in the behavior sphere. We reason that need-satisfaction in the interface and task spheres from digital learning tools will be manifested as higher engagement, learning, and well-being.

Given that some technologies may be too need-satisfying in the interface sphere (games, social media, *etc*.), which may lead to overuse (e.g., Rigby and Ryan, 2011; Peters et al., 2018), it is important to include well-being in the life sphere in order to rule out any canceling effect that need-satisfaction in the interface and task spheres might have on students' overall lives. To account for this, we measure flourishing because it is a measure of “the optimal end of human psychological functioning” (Calvo and Peters, 2014) and in line with the eudaimonic perspective of psychological well-being within SDT (Deci and Ryan, 2008). However, due to differences in digital learning tools and how they relate to life and well-being, we only expect our model to account for small amounts of variation in well-being. Lastly, we expect that need-satisfaction in the interface sphere will positively and indirectly predict user engagement, learning, and well-being. These expectations are in accordance with the above-mentioned research on METUX by Peters et al. (2018) and supported by theoretical assumptions and empirical evidence by Ryan et al. (2006) and Peng et al. (2012).

## Materials and Methods

### Participants and Procedure

The participants comprised of 426 (54% females, *M*_age_ = 23.73, SD = 4.09) higher education students in natural sciences (*n* = 147), social sciences (*n* = 109), economics (*n* = 94), health (*n* = 30), humanities (*n* = 24), law (*n* = 5), and arts (*n* = 14). Students who participated in this study were both bachelor's degree and master's degree students, and the average student had 3.44 years of higher education. The participants were drawn from two sub-samples. Sample 1 consisted of 224 (58.9% females, *M*_age_ = 24.32, SD = 3.79) Norwegian higher education students. The students from sample 1 were recruited when participating in different campus courses. Sample 2 consisted of 201 (48.2% females, *M*_age_ = 23.07, SD = 4.32) higher education students recruited through TurkPrime crowding source (Litman et al., 2017). TurkPrime is a tool used to access people *via* the Internet to participate in research projects. We made an explicit inclusion criterion that participants from TurkPrime had to be college/university students. Each student was invited to participate *via* SurveyMonkey and responded to the survey described below by means of their own smartphone/computer.

Following the research on passion (Vallerand, 2015), we asked the students to report a self-selected idiosyncratic digital learning tool which they use for learning purposes. We provided the students with examples ranging from LMS and mobile apps to social media platforms because a pilot study had revealed that students may misinterpret what was meant by a digital learning tool. The participants in our study provided more examples of digital learning tools than the ones we provided them with. Hence, we are confident that the students did not only choose the examples of digital learning tools provided to them. The students' idiosyncratic digital learning tools were classified as mobile app (*n* = 71), software (*n* = 105), online resource (*n* = 235), and others (*n* = 14).

The study was registered with the Norwegian Centre for Research Data (NSD), and we followed the ethical guidelines from NSD. The students in both samples were informed that they could withdraw at any time and that participation was voluntary and anonymous. The students in the Norwegian sample were given the possibility to win one of 10 gift cards if they provided their mobile number on a different questionnaire. This was done in order to ensure that the participants' mobile numbers were not linked to their responses. Students from the TurkPrime sample were paid 1 USD for participation. Answering the questionnaire took around 5–7 min to complete.

### Measurements

#### Interface Need Satisfaction

To measure the students' interface satisfaction of the need for autonomy and competence, we used the 10-item Technology-Based Experience of Need Satisfaction scale (Peters et al., 2018). The students were asked to reflect on their experiences of using the digital learning tools that they declared and to rate their agreement on a Likert scale ranging from 1 (completely disagree) to 5 (completely agree). An item example for competence is “I feel very capable and effective at using the digital learning tool” and for autonomy “The digital learning tool provides me with useful options and choices.” We created two composite subscales measuring interface competence and interface autonomy. Cronbach's alpha has acceptable values for interface competence (α = 0.82) and interface autonomy (α = 0.74).

#### Task Need Satisfaction

We used the eight-item Technology-Based Experience of Need Satisfaction scale (TENS-Task; Peters et al., 2018). The students were asked to reflect on their experience of using the digital learning tool that they declared, and how they use it to learn. The students were asked to respond on a five-point Likert scale ranging from 1 (completely disagree) to 5 (completely agree). An item example for competence was “I feel confident in my ability to learn with the digital learning tool” and for autonomy was “I only use the digital learning tool to learn because I have to” (reversed coding). Two composite subscales were created to tap into task competence and task autonomy. Cronbach's alpha for these subscales is acceptable for task competence (α = 0.77) and task autonomy (α = 0.77).

#### User Engagement

To measure students' user engagement within their self-selected digital learning tool, we used the short User Engagement Scale (O'Brien et al., 2018). The scale consists of four subscales: focused attention (feeling absorbed in the interaction and losing track of time), perceived usability (negative affect experience as a result of the interaction and the degree of control and effort expended), aesthetic appeal (attractiveness and visual appeal of the interface), and reward (feeling investment or reward in the experience). The total scale consists of 12-items, with four items measuring each subscale. The students were asked to respond on a five-point Likert scale ranging from 1 (completely disagree) to 5 (completely agree). Item examples for each subscale were as follows: “I lose myself in the digital learning tool when I use it” (focused attention), “I feel frustrated when I use the digital learning tool” (perceived usability), “The digital learning tool is attractive” (aesthetic appeal), and “Using the digital learning tool is worthwhile” (reward). Cronbach's alpha is acceptable for focused attention (α = 0.88), perceived usability (α = 0.87), aesthetic appeal (α = 0.83), and reward (α = 0.80).

#### Perceived Learning

We employed an adapted four-item scale to measure the students' perceived learning when using their self-selected digital learning tool. The scale was an adapted version used by Jeno et al. (2019a,b) to measure perceived learning among a higher education sample. The students were asked to respond on a seven-point Likert scale ranging from 1 (not true at all) to 7 (completely true). An item example is “By using the digital learning tool, I have learnt more than usual.” A composite score was created, and Cronbach's alpha for the scale is α = 0.86.

#### Well-Being

To measure the students' well-being, we used the eight-item flourishing scale (Diener et al., 2010). In line with the METUX model, well-being was measured at a general level and not referenced to the students' self-selected digital learning tool. The students were asked to respond on a seven-point Likert scale ranging from 1 (strongly disagree) to 7 (strongly agree). A composite score was created, where higher values indicate a student with many psychological resources and strengths. An item example is “I lead a purposeful and meaningful life.” Cronbach's alpha is acceptable (α = 0.93).

### Plan for Analysis

All analyses were conducted with the statistical program R version 3.6.2 (R. Core Team, 2018). Data preparation, descriptive analyses, and correlational analyses were conducted in the R-packages “car” (Fox and Weisberg, 2011), “memisc” (Elff, 2019), “multicon” (Sherman, 2015), “AutoModel” (Lishinski, 2015), and “psych” (Revelle, 2018). Confirmatory factor analyses (CFA) and structural equation modeling (SEM) were conducted with the R-packages “Lavaan” (Rosseel, 2012) and “semTools” (Jorgensen et al., 2020), with “semPlot” (Epskamp, 2019) for visualization.

Model fit for the CFA and SEM was assessed by means of conventional indices. Comparative Fit Index (CFI), Tucker–Lewis Index (TLI), and Incremental Fit Index (IFI) above or equal to 0.90, and root mean square error of approximation (RMSEA) and standardized root mean square residual (SRMR) within 0.00–0.05 is recommended, whereas 0.05–0.10 is acceptable (Schermelleh-Engel et al., 2003; Kline, 2011). Convergent validity of the scales was assessed by evaluating the average variance extracted (AVE) which is recommended to be above 0.5, while the evaluation of standardized loading on the latent variables is recommended to be minimally >0.5, but ideally >0.7 (Hair et al., 2009). Finally, model re-specification was done through evaluation of a large Lagrange multiplier which reflects how much the overall model chi-square value would decrease if that particular parameter was estimated in a subsequent model re-specification (Byrne, 2016). Missing data were handled *via* the full information maximum likelihood (FIML) approach. The FIML approach is, according to Byrne (2016), the recommended approach for handling missing data because it is the least biased approach.

## Results

### Descriptive Results

The descriptive analyses for each of the study variables are presented in Table 1. The descriptive analyses show acceptable standard deviations, skewness, and kurtosis values suggesting normality. The analyses of the normality assumptions on the dependent variables show no violation of normality. Correlates between the study variables (Table 2) show that interface competence and interface autonomy are positively related to all variables except focused attention, to which they are all negatively related. Similar results are found for task competence and task autonomy; however, these variables are unrelated to focused attention.

**Table 1:** Descriptive statistics for the total sample.

**Variables**	***n***	**Mean**	**SD**	**Min**	**Max**	**Range**	**Skew**	**Kurtosis**	**SE**
Interface competence	426	4.06	0.79	1.40	5.00	3.60	−0.86	0.22	0.04
Interface autonomy	426	4.08	0.72	1.20	5.00	3.80	−0.82	0.31	0.03
Task competence	425	4.15	0.77	1.25	5.00	3.75	−0.86	0.26	0.04
Task autonomy	425	2.50	1.17	1.00	5.00	4.00	−0.44	−0.61	0.05
Focused attention	426	3.48	0.64	1.42	5.25	3.83	0.30	−0.92	0.06
Perceived usability	426	3.96	1.01	1.00	5.00	4.00	−0.86	−0.03	0.05
Aesthetic appeal	426	3.47	0.93	1.00	5.00	4.00	−0.47	0.16	0.04
Reward	426	3.98	0.78	1.00	5.00	4.00	−0.84	1.03	0.04
Perceived learning	425	4.78	1.39	1.00	7.00	6.00	−0.34	−0.34	0.07
Well-being	424	5.70	1.10	1.00	7.00	6.00	−1.11	1.35	0.05

**Table 2:** Means, standard deviations, and correlations with confidence intervals of the variables of the total sample.

**Variables**	**1**	**2**	**3**	**4**	**5**	**6**	**7**	**8**	**9**
**Interface competence**									
Interface autonomy	0.56^**^								
	[0.49,0.62]								
Task competence	0.72^**^	0.61^**^							
	[0.68,0.77]	[0.55,0.67]							
Task autonomy	0.48^**^	0.54^**^	0.54^**^						
	[0.41,0.55]	[0.47,0.60]	[0.47,0.61]						
Focused attention	−0.13^**^	−0.14^**^	−0.08	−0.08					
	[−0.23, −0.04]	[−0.23, −0.04]	[−0.18,0.01]	[−0.17,0.01]					
Perceived usability	0.73^**^	0.59^**^	0.69^**^	0.54^**^	−0.22^**^				
	[0.68,0.77]	[0.52,0.65]	[0.63,0.73]	[0.47,0.61]	[−0.31, −0.13]				
Aesthetic appeal	0.21^**^	0.21^**^	0.27^**^	0.21^**^	0.33^**^	0.26^**^			
	[0.11,0.30]	[0.12,0.30]	[0.18,0.36]	[0.12,0.30]	[0.24,0.41]	[0.17,0.35]			
Reward	0.23^**^	0.40^**^	0.36^**^	0.27^**^	0.34^**^	0.27^**^	0.57^**^		
	[0.14,0.32]	[0.31,0.47]	[0.27,0.44]	[0.18,0.36]	[0.26,0.43]	[0.18,0.35]	[0.51,0.63]		
Perceived learning	0.23^**^	0.37^**^	0.41^**^	0.25^**^	0.30^**^	0.25^**^	0.40^**^	0.64^**^	
	[0.14,0.32]	[0.28,0.45]	[0.33,0.49]	[0.16,0.34]	[0.21,0.38]	[0.16,0.34]	[0.32,0.48]	[0.58,0.69]	
Well-being	0.19^**^	0.21^**^	0.20^**^	0.14^**^	0.04	0.16^**^	0.13^**^	0.22^**^	0.14^**^
	[0.09,0.28]	[0.12,0.30]	[0.11,0.29]	[0.05,0.24]	[−0.05,0.14]	[0.07,0.25]	[0.03,0.22]	[0.13,0.31]	[0.05,0.23]

*Values in square brackets indicate the 95% confidence interval for each correlation*.

* p < 0.05;

** *p < 0.01*.

### Confirmatory Factor Analyses

In order to test the factor structure for each latent variable, we performed separate CFAs for each of the study variables. The results from the CFA for interface competence reveal a poor model fit: χ^2^ = 173.15(5), CFI = 0.81, TLI = 0.62, RMSEA = 0.28, and SRMR = 0.10. Modification indices suggest covarying items 1 and 2 (both reverse-worded). We re-specified the model, and the model fit improved substantially. Results from the chi-square difference test indicate that the new model is significantly better: χ^2^-diff = 156(1), *p* < 0.001. For interface autonomy, CFA shows a poor model fit: χ^2^ = 68.73(5), CFI = 0.90, TLI = 0.80, RMSEA = 0.17, and SRMR = 0.09. The model evaluation shows that two items, both negatively worded, have weak factor loadings (<0.30), and these items are therefore omitted. Finally, the CFA for task competence also shows a poor model fit: χ^2^ = 88.35(2), CFI = 0.85, TLI = 0.55, RMSEA = 0.31, and SRMR = 0.09. The modification indices suggest that the covariation between items 1 and 2 (both reverse-worded) would improve the model fit. Re-specification of the model with covariation between items 1 and 2 improved the model fit significantly: χ^2^-diff = 81.6(1), *p* < 0.001. The model fit for the remaining latent variables is within the acceptable range and as expected. We find that the standardized factor loadings for each scale are within the acceptable range for most scales. One item has a standardized factor loading of 0.47 and 0.58 for interface competence and perceived learning, respectively, which is below the recommended range. Furthermore, AVE for interface autonomy, task competence, and task autonomy are below the recommended threshold, indicating that more error remains than can be explained by the latent factors of the items. The final results of the CFA for each latent variable are presented in Table 3. We also performed a measurement model where we included all of the latent variables along with their items to investigate how well the overall measurement model fits the data. The results show that, after modifying our model in accordance with the above-mentioned CFA for each individual latent variable, the measurement model has an acceptable model fit: χ^2^ = 1,014.76(333), CFI = 0.90, TLI = 0.89, RMSEA = 0.06 (0.06, 0.07), and SRMR = 0.07.

**Table 3:** Confirmatory factor analysis of all the study variables.

**Model**	**χ^**2**^(df)**	**CFI**	**TLI**	**RMSEA (95% CI)**	**SRMR**	**AVE**	**Standardized factor loadings**
Interface competence	17.03(4)^**^	0.98	0.96	0.08 (0.04, 0.13)	0.02	0.51	0.47–0.83
Interface autonomy	0.0(0)	1.00	1.00	0.00 (0.00, 0.00)	0.00	0.47	0.68–0.86
Task competence	6.73(1)^**^	0.99	0.94	0.11 (0.04, 0.20)	0.01	0.47	0.51–0.77
Task autonomy	41.82(2)^***^	0.91	0.75	0.21 (0.16, 0.27)	0.05	0.46	0.62–0.73
Engagement	188.36(48)^***^	0.94	0.82	0.08 (0.07, 0.09)	0.06		0.59–0.90
Perceived learning	5.49(2)	0.99	0.99	0.06 (0.00, 0.13)	0.01	0.64	0.48–0.92
Well-being	105.64(20)^***^	0.96	0.95	0.10 (0.08, 0.12)	0.03	0.63	0.74–0.85

*CFI, comparative fit index; TLI, Tucker–Lewis index; RMSEA, root mean square error of approximation; SRMR, standardized root mean square residual; AVE, average variation extracted; CI, confidence interval*.

*Engagement was specified as a four-factor model. AVE for the specific subscales where focused attention = 0.72, perceived usability = 0.69, aesthetic appeal = 0.63, and reward = 0.58*.

* p < 0.05;

** p < 0.01;

*** *p < 0.001*.

### Structural Equation Modeling

**Figure 2:**
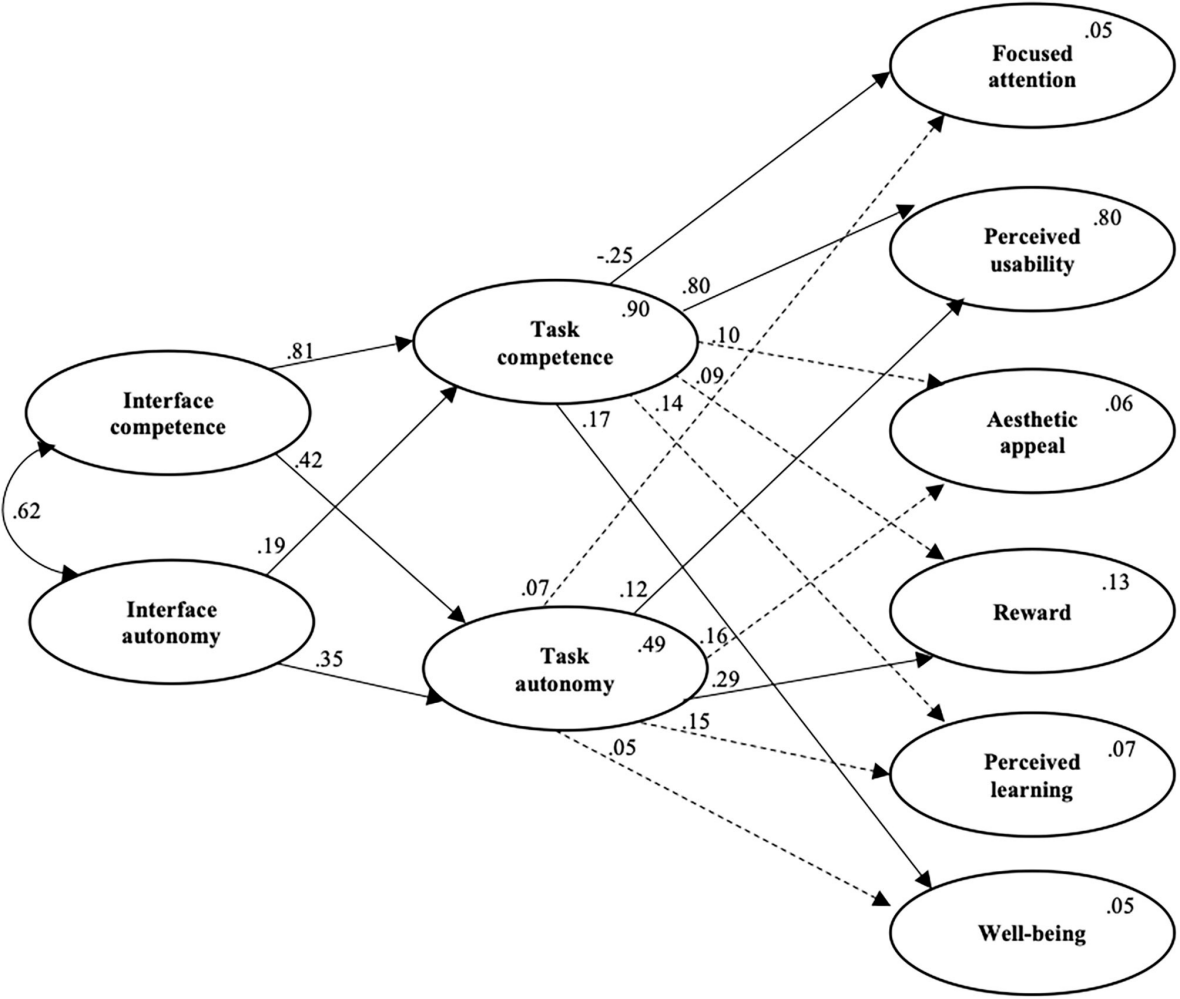
Final structural equation model. Results from the structural equation model (SEM). Values within the endogenous variables are the *R*^2^ values. Solid lines are significant coefficients (*p* < 0.05), whereas stippled lines are non-significant coefficients (*p* > 0.05). All values are standardized regression coefficients. For simplicity, we have removed the measurement model.

A full latent structural equation model was specified according to theoretical assumptions described in the introduction section. The overall results of the structural model (Figure 2) show an acceptable model fit: χ^2^ = 1,719.83(705), *p* < 0.001, CFI = 0.91, TLI = 0.90, IFI = 0.90, RMSEA = 0.05 (0.05, 0.06), and SRMR = 0.07. The model shows that interface competence and interface autonomy positively predict task competence. Task autonomy is positively predicted by both interface competence and interface autonomy. Task competence is negatively related to focused attention, whereas task autonomy is unrelated. Perceived usability is predicted by task competence but is unrelated to task autonomy. Task competence is unrelated to aesthetic appeal, whereas task autonomy is positively related. Task competence is unrelated to reward, whereas task autonomy is positively related. Perceived learning is unrelated to both task competence and task autonomy. Finally, task competence is positively related to well-being, whereas task autonomy is unrelated.

The indirect effect analyses (Table 4) show that nine of the 24 specified indirect effects are significant. Specifically, we find that interface competence indirectly and negatively predicts focused attention *via* task competence as mediator. Furthermore, the effect of interface competence increases perceived usability indirectly and positively *via* task competence. Interface competence positively enhances well-being through the effect of task competence. Interface competence indirectly and positively predicts perceived usability *via* task autonomy.

**Table 4:** Indirect effects analyses.

**Exogenous variable**	**Mediator**	**Endogenous variable**	***B***	**95% CI**
Interface competence	Task competence	Focused attention	−0.49^**^	−0.82, −0.15
Interface competence	Task competence	Perceived usability	1.48^***^	1.11, 1.85
Interface competence	Task competence	Well-being	0.41^*^	0.03, 0.79
Interface competence	Task autonomy	Perceived usability	0.11^*^	0.01, 0.22
Interface competence	Task autonomy	Reward	0.15^**^	0.04, 0.26
Interface autonomy	Task competence	Focused attention	−0.06^*^	−0.12, −0.01
Interface autonomy	Task competence	Perceived usability	0.19^***^	0.08, 0.29
Interface autonomy	Task autonomy	Perceived usability	0.05^*^	0.00, 10
Interface autonomy	Task autonomy	Reward	0.06^**^	0.01, 0.12

*Only significant indirect effects are shown. Values are shown as unstandardized regression coefficients*.

* p < 0.05;

** p < 0.01;

*** *p < 0.001*.

Interface autonomy predicted both focused attention negatively and perceived usability positively through the effect of task competence as mediator. Interface autonomy is indirectly and positively related to perceived usability, mediated through task autonomy. Lastly, the effect of interface autonomy on reward is mediated by task autonomy.

## Discussion

The main aim of this study is to investigate the extent to which the design of digital learning tools in the interface and task spheres predict engagement, learning, and well-being among students in higher education. We tested these relations on the basis of the METUX model which is derived from SDT. In general, the results from our analyses partly support our hypotheses.

As expected, we find support for the assumption that need-satisfaction in the interface sphere predicts need-satisfaction in the task sphere. This is found both at the correlational level and in our structural equation model. Importantly, the shared variance between need-satisfaction in the interface and task spheres is not substantially high, indicating that we are measuring two distinct constructs (i.e., spheres). The strongest coefficient in our model is the effect from interface competence to task competence which shares ~66% of the variance. These findings are in line with the preliminary results of Peters et al. (2018) and hint at the importance of need-balance between the spheres. As suggested by the METUX model, it is not sufficient that digital learning tools have need-satisfying features in the interface sphere if there is no need-satisfaction in the task and behavior spheres. Furthermore, imbalance of need-satisfaction in the interface sphere may account for overuse, but not efficient learning or well-being in the behavior and life spheres, respectively. This is suggested by SDT (Ryan and Deci, 2017; Peters et al., 2018) and has been supported by empirical research investigating need-balance across domains on psychological well-being (Milyavskaya et al., 2009).

We find only partial support for our predictors of engagement, learning, and well-being. Specifically, we find that task competence is a negative predictor of focused attention, while interface competence is a negative and indirect predictor, which is unexpected. However, task autonomy has no effect on engagement, learning, and well-being. This latter finding is also unexpected, given that need-satisfaction within technology should enhance feelings of immersion as suggested by the METUX model (Peters et al., 2018) and shown in previous studies (Ijaz et al., 2020). An explanation for this lack of a relationship between task autonomy and engagement, learning, and well-being could be that we investigated the use of digital learning tools for learning purposes. Hence, the experience of immersion is less salient and may even be detrimental for learning. For instance, students collaborating on a project through social media platforms may be more concerned about completing a specific learning task than about being immersed in a particular learning platform.

For perceived usability, both task competence and task autonomy are positive predictors, while interface competence and interface autonomy are indirect predictors. This is in line with the METUX model which suggests that need-satisfaction at both the interface and task level will increase perceived usability (Peters et al., 2018). This effect is explained by mechanisms such as novelty, dense and clear feedback, and choices (Rigby and Ryan, 2011).

We find no support for the effect of task competence or task autonomy on aesthetic appeal, which is unexpected. An explanation for this finding could be that attractiveness and appealing design, which are elements of the aesthetic appeal, are less “inward” focused and more “outward” focused and thus less need-satisfying (Ryan et al., 1996; Vansteenkiste et al., 2006). However, aesthetics may also facilitate the learning process through increased clarity, comprehension, emotions, and communication (Peters, 2014), which may be experienced as competence supportive. More research is needed to disentangle these different dynamics within technology and digital learning design.

Regarding the experience of learning as a reward, only task autonomy is a positive predictor. However, both interface competence and interface autonomy are positive indirect predictors and affect reward through the effect of task autonomy. This is in line with what could be expected by SDT, as the learning experience might feel rewarding because you choose the behavior, you feel agency in the digital learning tools, or you received effectance-relevant feedback in order to achieve a goal, for instance (Rigby and Ryan, 2011; Peters, 2014).

Unexpectedly, neither task competence nor task autonomy is a significant predictor of perceived learning. Despite the correlation matrix showing positive correlations between perceived learning and the predictor variables, when competing for variance, the effect disappears. This is surprising given that previous studies have found support for the satisfaction of basic psychological needs and learning (Fathali and Okada, 2017; Yang et al., 2018). Moreover, SDT would suggest that applying skills, feeling efficacious, receiving feedback, and structure are necessary conditions for growth and learning (Ryan and Deci, 2017). In terms of autonomy, there might be differences within the digital learning tools to the extent that they provide meaningful choices at different levels that would truly constitute autonomy satisfaction. Future studies would need to investigate if these results are replicated in a similar context. It could be, for instance, that there are other underlying mechanisms satisfying the needs for competence and autonomy that are not currently measured in the scales.

As expected, our model is only able to explain small amounts of variance in psychological well-being. This effect is mainly accounted for by task competence and the indirect effect of interface competence. Our operationalization of well-being is measured through the concept of flourishing, which has been proposed as a candidate construct by Peters et al. (2018). A construct such as flourishing is in line with SDT's conceptualization of eudaimonic well-being (Deci and Ryan, 2008). However, it is possible that our model could have explained more variance in psychological well-being if we had conceptualized it as hedonic well-being. Happiness and life satisfaction are considered indicators of subjective well-being or hedonic well-being (Kahneman et al., 2006; Diener et al., 2017), which suggests that well-being is the presence of a positive affect and the absence of a negative affect. In terms of digital learning tools, it is possible that some students experience more or less positive and negative affect within their self-selected digital learning tools. Experiences of hedonic well-being might be due to need-satisfaction in the different spheres or other factors such as reward systems or large amounts of positive emotions within the digital learning tools. This, in turn, might account for more explained variance in hedonic well-being than in eudaimonic well-being. It would be interesting, in future studies, to include both types of indicators of well-being in order to disentangle the effects of the sphere on eudaimonic and hedonic well-being.

### Limitations

There are several limitations worth mentioning before interpreting the results from our study. First, this is a correlational study, and one should be careful to draw any causal inferences from the results. We recommend future studies testing the validity of the METUX model in a longitudinal design to evaluate how need-satisfaction within learning tools can enhance engagement, learning, and well-being over time.

Second, we did not include all spheres from the METUX model. It could be interesting to include the adoption sphere and investigate whether autonomous or controlled reasons for adoption of a specific tool impact the engagement, learning, persistence, or well-being of students. Specifically, by including a measure of the relative autonomous reason for adopting or using a digital learning tool, we could have understood the underlying mechanisms of our outcome measures. For example, some students may use Coursera because they find it enjoyable or important to do so when learning new knowledge (autonomous motivation). In contrast, some students may use Coursera because they feel pressured by teachers or because they want to show that they are intelligent compared to other peers (controlled motivation). These different underlying reasons for using digital learning tools may have an impact on behavioral engagement, persistence on task, and learning. Hence, we recommend future studies to measure the adoption sphere. Similarly, if measuring relevant digital learning tools, future studies would have to include the social sphere in order to further test the validity of the METUX model. Furthermore, we did not measure relatedness. This could be important given that relatedness satisfaction may be crucial when students are collaborating through social media, for instance. Future studies would need to replicate our study and include relatedness in their model. In order to remove the confounding effect of relatedness found in different digital learning tools, future studies should include only social media platforms in order to test the effect of relatedness on engagement, learning, and well-being.

Third, we only measured perceived learning as opposed to actual achievement. It may be argued that actual academic achievement scores are more reliable than perceived learning (Kuncel et al., 2005; Felder-Puig et al., 2012), despite some overlap. However, the design of the present study prevented the inclusion of formal academic achievement scores. We recommend future studies to be designed so that formal academic achievement scores can be included.

Lastly, regarding sample size and drawing samples from Norway and TurkPrime, although we tried to have samples drawn from different contexts (i.e., Norway- and US-based college/university students), future studies need to investigate these relations in multiple countries (cross-cultural) and contexts (elementary, upper secondary). This will further test the validity of the METUX model.

### Theoretical and Practical Considerations

Several implications can be drawn from our results. First, our confirmatory factor analyses show that some of the items on specific scales need improvement. Specifically, we find that negatively worded items are the items that were removed or needed residual covariation, both of which are recommended with caution (DeVellis, 2017). Moreover, results from the average variance extracted test suggest that some scales have lower-than-suggested convergent validity. Future studies would need to conduct more psychometric evaluations of the scales in order to validate their usefulness in the digital learning context. It would also be important to conduct measurement invariance tests across gender, age, and technologies, to investigate if the scales work similarly across these factors. This would be in line with the general tenets of SDT which suggest that the satisfaction of basic psychological needs should function across contexts, gender, and developmental age groups (Ryan and Deci, 2017). Given our present results, we suggest item and scale improvement on some of the scales (i.e., interface autonomy, task competence, task autonomy) due to low AVE. Fornell and Larcker (1981) argue that AVE may be more conservative than reliability measures as indicators of convergent validity. Thus, future scale development should identify which items show lower inter-item correlation, low item AVE, and low factor loadings for further scale improvement.

Second, the results of this study show the importance of testing user engagement as a multidimensional construct and not as an aggregate construct (O'Brien, 2016). For instance, our results show a differential effect of task competence and autonomy on the different user engagement subscales. Specifically, in line with SDT, it may be more need-satisfying to have digital learning tools that are high on usability, as opposed to aesthetically appealing. Thus, if used as a composite construct, these differential effects might be canceled out. We recommend that future studies using a multidimensional construct of user engagement do not combine the different factors into a general user engagement measure. An interesting future line of research is to measure the psychological construct of engagement, as measured through behavioral, emotional, cognitive, and agentic engagements (Reeve, 2012), that is, it would be interesting to understand the extent to which need-satisfaction within the different spheres can account for the active involvement in a learning activity as engagement is defined.

Finally, we recommend future studies to investigate the validity of the model within a domain- and situation-specific context. We investigated the METUX model within a higher education context, albeit across several courses, and with self-selected idiosyncratic digital learning tools. In contrast, it could be beneficial to investigate students' engagement, learning, and well-being on a specific course where there is use of a learning management system for situation-specific course assignments or tasks. This could allow for less error and a more nuanced interpretation of the investigated model. Such an analysis would allow us to understand the underlying need-supportive elements in the course, assignments, or tasks in the digital learning tool and its need-satisfying experiences in the different spheres. This would further strengthen the validity of the METUX model.

The implications of our results can potentially be important for technology designers and end-users (i.e., students). The METUX model can potentially be used to inform different digital learning tools, learning management systems, and design of motivational mechanisms to facilitate engagement, learning, and well-being. However, there is a need for more empirical research to further test the validity of the METUX model in different contexts by using a different research design before implementing this as a framework for designing digital learning tools in educational contexts.

## Data Availability Statement

The dataset generated for this study is available in the Open Science Framework repository [https://osf.io/ds2pu/]. R-codes for the analyses are available in the GitHub repository [https://github.com/lujeno/METUX/blob/main/METUX2018.R].

## Ethics Statement

The studies involving human participants were reviewed and approved by Norwegian Centre for Research Data. Written informed consent for participation was not required for this study in accordance with the national legislation and the institutional requirements.

## Author Contributions

LJ and J-AG designed the study. LJ and ÅD prepared the study materials. LJ collected and analyzed the data and wrote the first draft of the manuscript. All the authors have read, commented, and approved the submitted version.

## Conflict of Interest

The authors declare that the research was conducted in the absence of any commercial or financial relationships that could be construed as a potential conflict of interest.
